# Characteristics of the gut microbiota in pregnant women with fetal growth restriction

**DOI:** 10.1186/s12884-022-04635-w

**Published:** 2022-04-07

**Authors:** Xinzhi Tu, Chun Duan, Bingying Lin, Kangfeng Li, Jie Gao, Huaying Yan, Kejian Wang, Zhao Zhao

**Affiliations:** 1 Department of Obstetrics and Gynecology, Affiliated Shenzhen Maternity & Child Healthcare Hospital, Southern Medical University, Shenzhen, China; 2 Clinical laboratory, Affiliated Shenzhen Maternity & Child Healthcare Hospital, Southern Medical University, Shenzhen, China; 3 The Third Affiliated Hospital of Shandong First Medical University (Affiliated Hospital of Shandong Academy of Medical Sciences), Jinan, China; 4 Gastroenterology Institute and Clinical Center of Shandong First Medical University, Jinan, China; 5 Department of Anesthesiology, Shenzhen University First Affiliated Hospital / Shenzhen Second People’s Hospital, Shenzhen, China

**Keywords:** Fetal growth restriction (FGR), Gut microbiota, 16S rDNA sequencing, Pregnant women, Glycometabolism

## Abstract

**Background:**

Fetal growth restriction (FGR) in utero leads to failure of fetus to reach the genetically normal growth potential. Currently available means of treating FGR are limited. And it remains unknown how pregnant women who give birth to FGR fetus differ in gut microbiota composition from normal pregnant women.

**Methods:**

In this case-control study, fecal samples were obtained from maternal rectum in the operation room by an obstetrician under strict aseptic conditions. We compared gut microbiota of 14 pregnant women with FGR and 18 normal controls by performing 16S rDNA amplicon sequencing.

**Results:**

We identified significant differences in β-diversity between the FGR and control groups (*P* < 0.05). At genus level, *Bacteroides*, *Faecalibacterium* and *Lachnospira* were highly abundant in the FGR subjects, which are significantly enriched in Kyoto Encyclopedia of Genes and Genomes (KEGG) pathways related to glycometabolism.

**Conclusion:**

These findings demonstrated that the distinct composition of the gut microbiota between FGR and normal pregnant women could contribute to an improved understanding of the prevention and treatment of FGR.

## Background

Fetal growth restriction (FGR), also known as intrauterine growth restriction/retardation (IUGR), is a pathologic condition that is defined as the fetus failing to achieve its genetically predetermined growth potential [1]. It has an increased risk of perinatal morbidity and mortality [2, 3], which also leads to cardiovascular and metabolic diseases such as diabetes and obesity in later life [4, 5]. Currently available means of treating FGR in utero are limited, antenatal recognition and appropriate maternal-fetal managements can help choose the optimal time of delivery and improve perinatal outcome [6, 7].

Human gut microbiota plays a unique part in metabolism, immunity, and nutrition absorption [8]. A variety of studies on pregnant women have identified a link between changes in fecal bacterial abundance and the pathogenesis of certain disorders, such as gestational diabetes mellitus (GDM), preeclampsia (PE), maternal obesity [9–12]. More encouragingly, probiotic supplements might be an assistant treatment strategy for these complications. A systematic review which included a total of 20 randomized controlled trials involving 2972 participants found that probiotic supplements had certain functions to reduce the level of fasting plasma glucose (FPG) and improve insulin, insulin resistance, and insulin sensitivity, especially for GDM and healthy pregnant women [13]. The long-term risk of growth-restricted fetuses is similar to that of offspring of women with GDM, and the role of insulin resistance has been recognized [14]. Numerous cohort studies and epidemiological studies in human populations suggest that the effects of GDM, PE, maternal obesity on intrauterine growth disturbances (both FGR and macrosomia) [15, 16]. Interestingly, a study using piglets as an experimental model indicated that IUGR significantly impairs small intestine structure, modifies gut microbiota colonization, and disturbs inflammatory and metabolic profiles during the first 12 h after birth [17]. The latest study found placental microbial composition significantly altered in neonates with FGR, while Neisseriaceae may constitute promising therapeutic targets for FGR treatment [18]. However, many gaps in knowledge remain as the difference in gut microbiota composition during pregnancy between FGR and normal pregnant women.

Here, we performed a case-control study using high-throughput 16S rDNA gene sequencing. The purpose of the present study was to characterize altered maternal gut microbiota in pregnant women with FGR, and to explore the role of these changes in the development of FGR.

## Materials and methods

### Study subjects

This study was approved by the Ethics Committee of Shenzhen Maternity & Child Healthcare Hospital on 20th February 2019. (No. SFYLS [2019] 062). All participants were made aware of the details of the study before obtaining written informed consent. After delivery, clinical data was extracted from medical records. From June 2019 to April 2020, singleton pregnant women who delivered by elective Cesarean section prior to labor were enrolled in this study at the Shenzhen Maternity & Child Healthcare Hospital. The indications for C-section were only restricted with advanced maternal age, abnormal presentation and repeated Cesarean section.

The inclusion criteria of the FGR group were as follows: 1) an estimated fetal weight (EFW) < 3th percentile for gestational age (GA) within 7 days of birth; 2) birth weight < 10th percentile; 3) placental disorders or umbilical cord abnormalities by postnatal confirmation. Meanwhile, the healthy controls were those with EFW between 25th to 90th percentile and birth weight between 10th to 90th percentile. The birth weight curve used in this study was based on data from 342 Asian women published by the National Institute of Child Health and Human Development [19]. GA was determined by the last menstrual period and confirmed by ultrasound in the first trimester.

None of the women in either group had: 1) maternal medical conditions except above mentioned indications for C-section; 2) fetal or infantile anomalies; 3) premature rupture of membranes; 4) infectious diseases; 5) preoperative fasting< 8 h; 6) alcohol or substance abuse; 7) any antibiotic exposure before stool collection (All prophylactic antibiotics was administrated after cutting umbilical cord). In total, 32 pregnant women involving the final analysis were divided into FGR group (*n* = 14) and the control group (*n* = 18).

### Maternal blood sample collection and measurement

A fasting venous blood sample (2 mL) was drawn within 3 days before C-section and centrifuged at 3000 rpm for 10 min to separate serum for the measurements. For accurate quantification of glucose and insulin, the blood sample was delivered to the laboratory within 2 h, and was measured within 6 h after centrifugation. Plasma glucose was measured by glucose oxidase method using Beckman Coulter UniCel DXC 800 Synchron™ Clinical Systems. Plasma insulin was measured by chemiluminescent enzyme immunoassay using Beckman Coulter DxI-800 analyzer.

### Fecal sample collection and DNA extraction

The first author as a senior obstetrician with 16 years of experience collected all fecal samples after anesthesia and before C-section in operation room under strict aseptic conditions and a uniform protocol. After disinfecting the anus with iodophor twice, a sterile Nylon flocked swabs (CY-98000, HCY Technology, Shenzhen, China) was gently inserted into the rectum (to a depth of 6 cm) and was rotated by 360°. Then, the swab tip was snapped off into a 1.5 mL sterile centrifuge tube containing preservation solution (CY-F002–10, HCY Technology, Shenzhen, China). These samples were immediately stored at − 80 °C until DNA extraction.

DNA from stool samples was extracted using Omega M5635–02 Kit according to manufacturer’s instructions. All experiments were carried out on super-clean table. The concentration and purity of DNA was quantified by NanoDrop 2000 spectrophotometer (Thermo Fisher Scientific, Wilmington, DE).

### 16S rDNA amplicon sequencing

PCR amplification of the bacterial 16S rRNA genes V3–V4 region was performed using the forward primer 341F (5′-CCTAYGGGRBGCASCAG-3′) and the reverse primer 806R (5′-GGACTACHVNNGGGTATCTAAT-3′). Sample-specific 7-bp barcodes were incorporated into the primers for multiplex sequencing. The PCR reaction volume was 25 μl. The PCR components contained 5 μl of 5× PCR buffer, 2 μl (2.5 mM) of dNTPs, 1 μl (10 uM) of Forward primer, 1 μl (10 uM) of Reverse primer, 1 μl of DNA Template, 0.25 μl of Fast pfu DNA Polymerase and 14.75 μl of ddH_2_O. Thermal cycling consisted of initial denaturation at 98 °C for 5 min, followed by 25 cycles consisting of denaturation at 98 °C for 30s, annealing at 55 °C for 30 s, and extension at 72 °C for 45 s, with a final extension of 5 min at 72 °C. PCR amplicons were purified with Agencourt AMPure Beads (Beckman Coulter, Indianapolis, IN) and quantified using the PicoGreen dsDNA Assay Kit (Invitrogen, Carlsbad, CA, USA). After the individual quantification step, amplicons were pooled in equal amounts, and paired-end 2 × 250 bp sequencing was performed using the Illlumina NovaSeq platform with NovaSeq 6000 SP Reagent Kit at Shanghai Personal Biotechnology Co., Ltd. (Shanghai, China). Raw sequencing data in this study were deposited into the NCBI’s Sequence Read Archive database (BioProject ID PRJNA820332).

### Bioinformatics and statistical analysis

Paired-end reads were assigned to samples based on their unique barcodes and were truncated by cutting off the barcodes and primer sequences. The relative abundance for each bacterial level from phylum to genus was measured using QIIME pipeline. The Chao, Ace, Shannon and Simpson indexes were calculated to assess ɑ-diversity within the group. The β-diversity was assessed by Principal coordinate analysis (PCoA) of unweighted Unifrac distance matrix and visualized by Non-metric multidimensional scaling (NMDS) plot. The linear discriminant analysis effect size (LEfSe) tool was used to identify taxa which could display significant differences in the two groups. The Phylogenetic Investigation of Communities by Reconstruction of Unobserved States (PICRUSt) computational approach was used to predict the biological functions of the differentially abundant taxa between two groups of samples [20]. PICRUSt highlighted the enriched functional categories of the KEGG (Kyoto Encyclopedia of Genes and Genomes) pathways [21]. Statistical analyses were performed using R software (version 3.6.1). Continuous variables were reported as means ± standard deviations. Student’s t-tests were used to study differences in continuous variables. *P*-value < 0.05 was considered statistically significant.

## Results

### Clinical information of subjects

A total of 14 pregnant women with FGR and 18 normal controls were included for final analysis. The clinical characteristics of all pregnant women are shown in Table 1. As expected, gestational age at birth and birth weight were significantly lower in FGR group than in control group (*P* < 0.01). There were no significant differences in maternal age, pregestational body mass index (BMI), maternal weight gain, fasting glucose and fasting insulin.

**Table 1:** Clinical information of subjects

	FGR Group (***n***=14)	Control Group (***n***=18)	***P***-value
Maternal age (year)	33.14 ± 4.63	32.67 ± 4.40	0.78
Pregestational BMI (kg/m^2^)	19.78 ± 1.51	21.19 ± 2.26	0.06
Maternal weight gain (kg)	13.31 ± 3.61	13.99 ± 3.92	0.63
Gestational age at delivery (week)	37.82 ± 0.95	39.20 ± 0.46	**1.2×10**^**-5**^
Birth weight (kg)	2.23 ± 0.21	3.28 ± 0.29	**5.4×10**^**-12**^
Fasting glucose (mmol/L)	3.90 ± 0.57	4.21 ± 0.40	0.11
Fasting insulin (pmol/L)	66.61 ± 44.68	68.74 ± 53.96	0.91

### Diversity of maternal gut microbiota

**Fig. 1:**
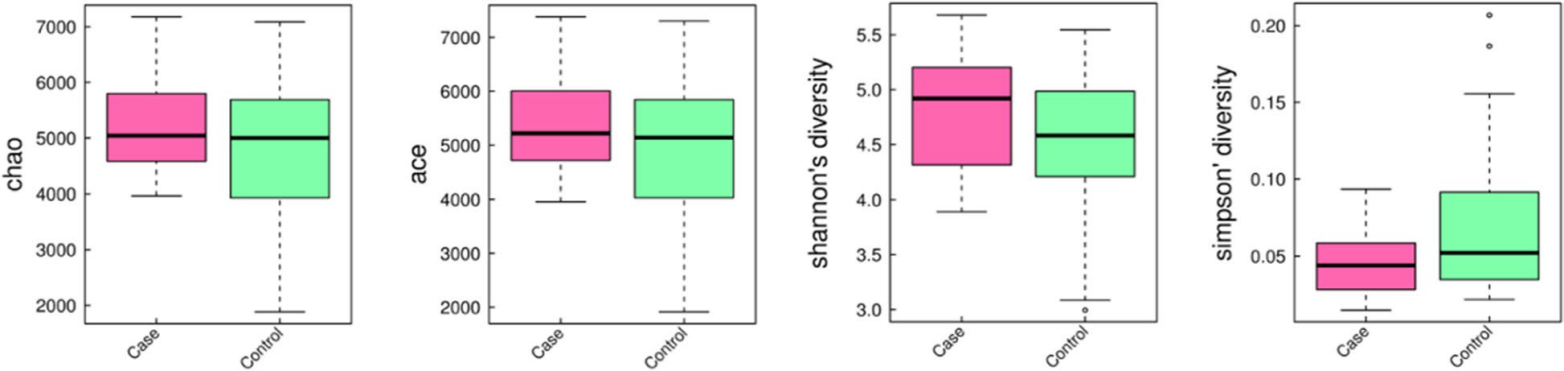
Comparison of α-diversity between the FGR and control groups. Four indexes were calculated to represent the α-diversity (**A** Chao index; **B** Ace index; **C** Shannon’s diversity index; **D** Simpson’s diversity index)

**Fig. 2:**
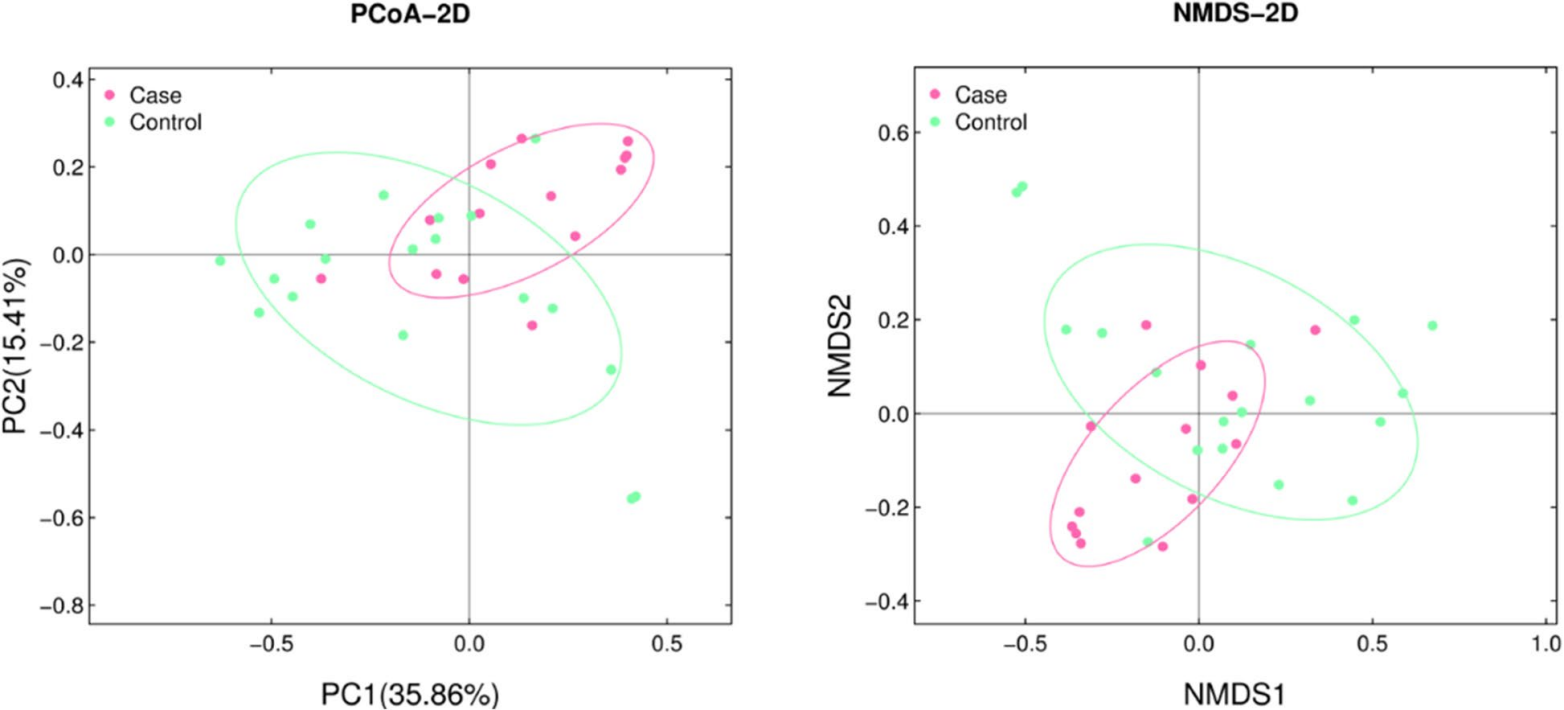
The separation of FGR and control samples based on the PCoA (**A**) and NMDS (**B**) according to the Bray-Curtis distance

To analyze the differences of gut microbiota between the two groups, 4,795,868 tags from 32 stool samples were obtained (average of 149,871 ± 22,842 tags per sample). All tags were clustered into 3849 operational taxonomic units (OTUs). The community richness of gut microbiota was evaluated based on α- and β-diversity in each sample. No significant differences in α-diversity represented by Chao, Ace, Shannon and Simpson indexes were found between two groups (*P* > 0.05, Fig. 1). On the other hand, the weighted UniFrac distance between individual samples was calculated to estimate the β-diversity in microbial communities (Fig. 2). Both PCoA and NMDS plots revealed that women with FGR tended to assemble and separate from the controls (*P* < 0.05).

### Differences in gut microbiota between two groups

**Fig. 3:**
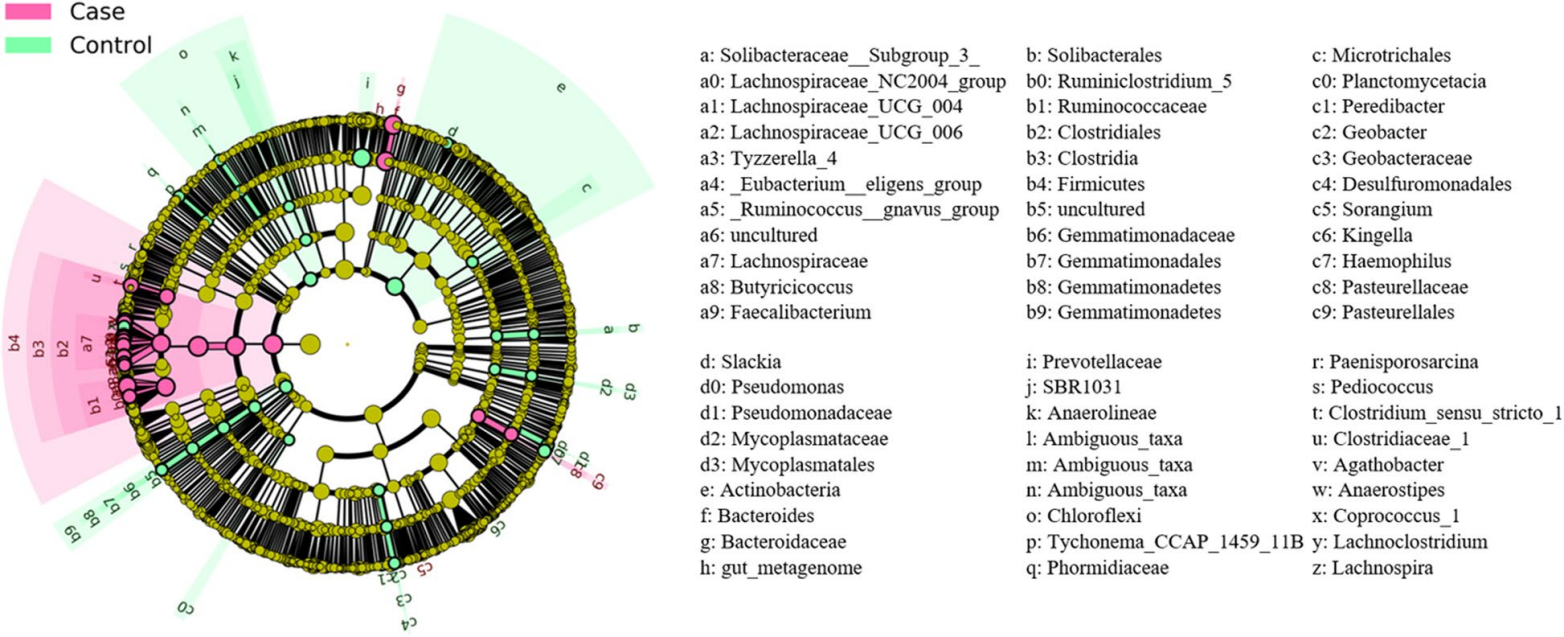
Cladogram of gut microbiota taxa between the FGR and control groups

The LEfSe analysis was used to identify differentially abundant taxa between FGR and control groups. (Fig. 3). At phylum level, *Firmicutes* was more abundant in the FGR group than in the control group. At genus level, we observed that *Bacteroides*, *Faecalibacterium*, *Lachnospira* (all belong to *Lachnospiraceae* family) were highly abundant in the FGR group as compared to the control group.

### Functional analysis of differentially abundant taxa

**Fig. 4:**
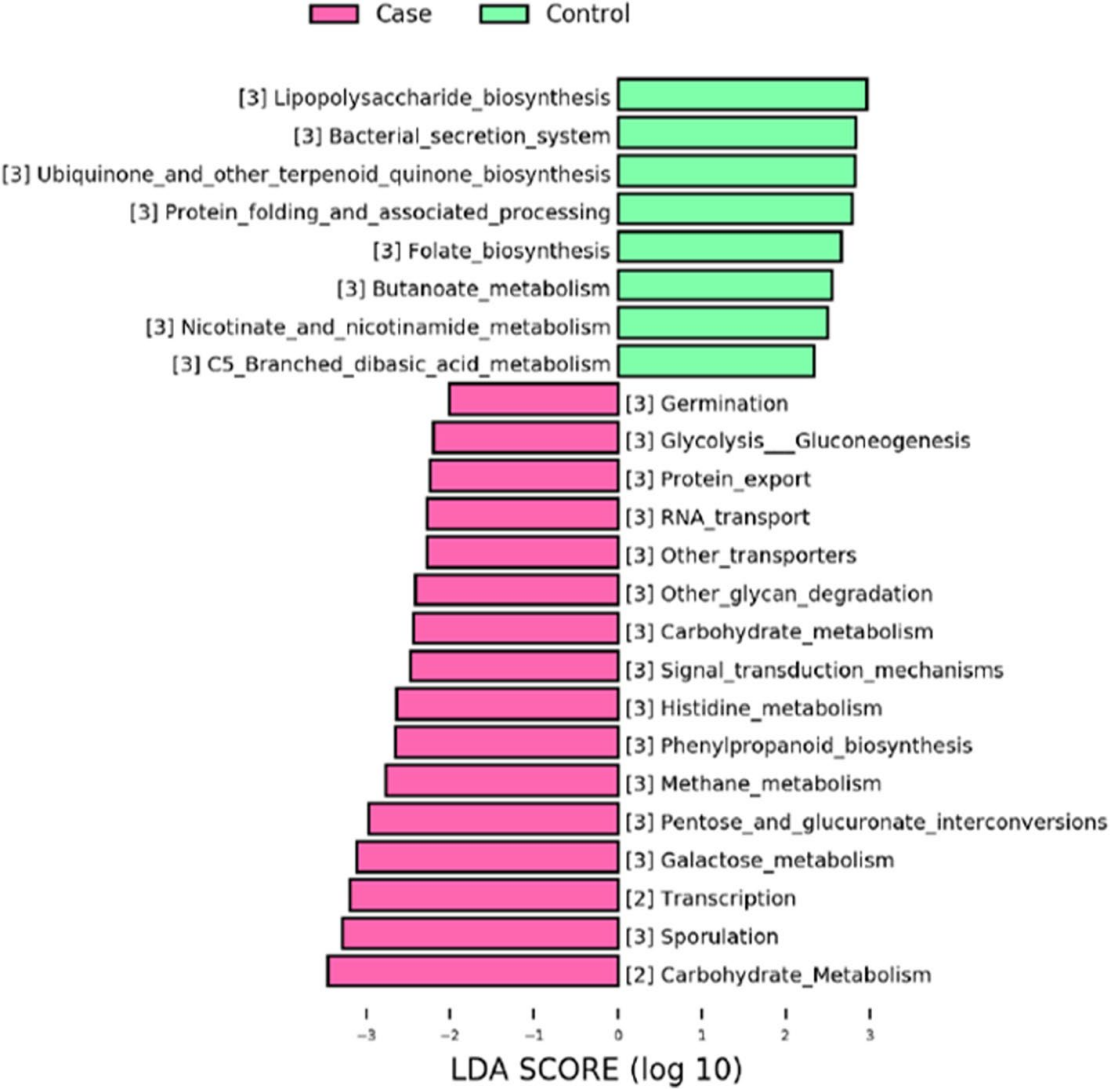
KEGG pathway analysis of differentially abundant microbial taxa based on PICRUSt software

To gain deeper insights into the relationship between FGR and gut microbiome functions, the PICRUSt software was implemented to predict the metabolic pathways potentially altered by dysbiosis (see Materials and Methods). The functional categories differentially enriched between the FGR and control groups were mainly involved glycometabolism (Fig. 4), including “Carbohydrate Metabolism”, “Glycolysis / Gluconeogenesis”, “Pentose and glucuronate interconversions” and “Galactose metabolism”. Together, these enriched pathways together suggested that FGR may alter the energy metabolism in the gut microbiota of pregnant women.

## Discussion

In this study, we demonstrated that the composition of maternal gut microbiota during pregnancy was significantly different between pregnant women with FGR and normal controls. The altered FGR-related microbial community was characterized by the increased abundance of genus *Bacteroides*, *Faecalibacterium*, *Lachnospira*. These findings might provide novel insight into the prevention and treatment of FGR.

Several indices including ACE, Chao, Shannon and Simpson were used to profile the maternal gut microbiota from different aspects. Despite the lack of significant difference in these indices, PCoA plot revealed complete segregation of the FGR and control group. Furthermore*,* the differential relative abundance of specific taxa was presented in the two groups. We found that the relative abundance of phylum *Firmicutes* was significantly higher in the FGR group than that in the control group, which was in keeping with the results of previous studies in pregnant women with GDM [8], pregestational overweight and obesity [22]. At genus level, *Bacteroides* was found to be increased in the FGR group. Previous studies demonstrated that increased *Bacteroides* was associated with overweight and obesity in both adults [23–25] and pregnant women [12], which could increase the risk of FGR and sudden intrauterine unexplained death [26, 27]. Moreover, in this study, *Faecalibacterium* and *Lachnospira* were also enriched in the FGR group. This is in agreement with results reported by Zacarias et al. that similar alterations were found in the overweight pregnant women compared to the normal ones [22]. In general, we found altered maternal microbiota in pregnant women with FGR, which was consistent with dysbiosis that occurred in various disorders during pregnancy.

It is well-known that obesity is associated with a state of chronic low-level inflammation [28]. Reactive oxygen species (ROS) production is elevated in obesity, which causes enhanced activation of inflammatory pathways [29, 30]. Interestingly, Xu et al. reported that ROS are involved in lipopolysaccharide (LPS)-induced intrauterine FGR in mice [31]. According to previous studies, a higher *Firmicutes/Bacteroidetes* ratio was associated with an aggravation of low-grade inflammation and to a more elevated capability of harvesting energy from food [32]. *Bacteroidetes*, a type of gram-negative bacteria, is the main contributor to LPS biosynthesis. Therefore, high abundances of *Bacteroidetes* may induce increased inflammation during pregnancy [33]. Maternal LPS exposure at late gestational stages results in intrauterine FGR in mice [31, 34]. A recent study indicated that the level of *Lachnospiraceae* correlated negatively with energy consumption and positively with leptin level [35]. In addition, Florencia et al. demonstrated inflammatory biomarker (high-sensitive C-reactive protein) values were correlated with several microbiota components, such as *Lachnospiraceae* and *Faecalibacterium* [22]. Taken together, the over-represented B*acteroides*, *Faecalibacterium* and *Lachnospiracea*e in FGR group might contribute to the development of FGR.

The greatest strength of our study is the homogenous characteristics of enrolled FGR cases. Placental disorders or umbilical cord abnormalities were the only causes of FGR among the participants, excluding maternal-fetal pathologies such as PE, diabetes, or fetal abnormalities. This reduced the confounding in microbiome data caused by heterogeneity in causes of FGR. In addition, an EFW below the third percentile was adopted as the threshold of diagnosis of FGR in our study, thus allowing us to avoid including constitutionally normal newborns. FGR is often confused with small for gestational age in clinical practice. And it is well known that lower growth percentile is associated higher likelihood of FGR and thus susceptibility to problems after birth [36]. Another strength is that we strictly controlled for sterile conditions during sampling. Considering that the fecal samples are usually expelled and collected in toilet, microbes may be contaminated during this process. In contrast, all samples in this study were directly obtained from maternal rectum in the operation room by the same senior obstetrician according to the principle of sterility, which minimized the possibility of microbial exposure and colonization in vitro.

However, several potential limitations need to be taken into consideration. Firstly, the sample size was relatively small and all the participants were recruited from the same hospital, thus we could not completely rule out the potential regional differences in maternal gut microbiota. The reliability of current results would greatly benefit from larger FGR and control cohorts. Secondly, we were not able to record detailed information on diet and lifestyle of the mothers during pregnancy, which have also been shown to alter the microbiome. Therefore, the associations of dietary intakes and the altered FGR-related microbial community were not analyzed in this study. Therefore, the mechanism by which alterations of maternal microbiome induce FGR should be further explored in animal experiments with well-controlled feeding conditions. Moreover, short-read 16S rDNA amplicon sequencing technique limited our ability to examine gut microbiota at species and strain level, which requires deeper taxonomic profiles from metagenomic shotgun sequences.

To our knowledge, this is one of the earliest studies to characterize the maternal gut microbiota in pregnant women with FGR. Our results indicated a relationship between maternal dysbiosis during pregnancy and the risk of FGR, which might involve the dysregulation of glycometabolism. Since gut microbiota profiles are alterable through various means (e.g., probiotics and dietary changes), our findings could provide novel insights into the prevention and treatment of FGR.

## Data Availability

The dataset supporting the conclusion of this article is available in the NCBI’s Sequence Read Archive database (BioProject ID PRJNA820332).

## Abbreviations

FGR: Fetal growth restriction
KEGG: Kyoto Encyclopedia of Genes and Genomes
IUGR: Intrauterine growth restriction / retardation
GDM: Gestational diabetes mellitus
PE: Preeclampsia
FPG: Fasting plasma glucose
EFW: Estimated fetal weight
GA: Gestational age
PCoA: Principal coordinate analysis
NMDS: Non-metric multidimensional scaling
LEfSe: Linear discriminant analysis effect size
PICRUSt: Phylogenetic Investigation of Communities by Reconstruction of Unobserved States
BMI: Body mass index
OTUs: Operational taxonomic units
ROS: Reactive oxygen species
LPS: Lipopolysaccharide
